# Immunohistochemical Analysis of Knee Chondral Defect Repair after Autologous Particulated Cartilage and Platelet-Rich Plasma Treatment in Sheep

**DOI:** 10.3390/ijms242015157

**Published:** 2023-10-13

**Authors:** Lourdes Alcaide-Ruggiero, Verónica Molina-Hernández, Juan Morgaz, J. Andrés Fernández-Sarmiento, María M. Granados, Rocío Navarrete-Calvo, José Pérez, Setefilla Quirós-Carmona, José M. Carrillo, Ramón Cugat, Juan M. Domínguez

**Affiliations:** 1 Departamento de Medicina y Cirugía Animal, Facultad de Veterinaria, Universidad de Córdoba, Hospital Clínico Veterinario, Campus de Rabanales, Ctra. Madrid-Cádiz Km 396, 14014 Córdoba, Spain; v92moroj@uco.es (J.M.); andres.fernandez.sarmiento@uco.es (J.A.F.-S.); pv2grmam@uco.es (M.M.G.); v92nacar@uco.es (R.N.-C.); v42quics@uco.es (S.Q.-C.); jmdominguez@uco.es (J.M.D.); 2 Fundación García Cugat para Investigación Biomédica, Plaza Alfonso Comín 5-7, 08023 Barcelona, Spain; jcarrillo@uch.ceu.es (J.M.C.); ramon.cugat@sporttrauma.com (R.C.); 3 Departamento de Anatomía y Anatomía Patológica Comparadas y Toxicología, UIC Zoonosis y Enfermedades Emergentes ENZOEM, Facultad de Veterinaria, Universidad de Córdoba, Edificio de Sanidad Animal, Campus de Rabanales, Ctra. Madrid-Cádiz Km 396, 14014 Córdoba, Spain; an1pearj@uco.es; 4 Departamento de Medicina y Cirugía Animal, Facultad de Veterinaria, Universidad CEU Cardenal Herrera, Hospital Clínico Veterinario, Calle Santiago Ramón y Cajal s/n, 46115 Valencia, Spain; 5 Instituto Cugat y Mutualidad de Futbolistas Españoles, Delegación Catalana, 08023 Barcelona, Spain

**Keywords:** chondral defect, knee, particulated cartilage, platelet-rich plasma, immunohistochemical

## Abstract

This study performs an analysis that will enable the evaluation of the quality, durability, and structure of repaired cartilaginous extracellular matrix tissue using an autologous-based particulated autograft cartilage and platelet-rich plasma treatment (PACI + PRP). A single-blind controlled experiment was conducted on 28 sheep to evaluate the efficacy of the PACI + PRP treatment for cartilage defects. Full-thickness 8 mm diameter defects were created in the weight-bearing area of both knees. The right knees received PACI + PRP. The left knees were treated with Ringer’s lactate solution (RLS) or hyaluronic acid (HA) injections. Sheep were euthanized at 9- or 18-months post-surgery. An extensive immunohistochemical analysis was performed to assess collagen types (I, II, III, V, VI, IX, X, XI) and aggrecan positivity. A semiquantitative scoring system provided a detailed evaluation of immunostaining. Collagens and aggrecan scores in the PACI + PRP groups were similar to healthy cartilage. Significant differences were found in collagens associated with matrix maturity (II and V), degradation (IX), structure and mechanics (VI), and hypertrophy (X) between healthy cartilage and RLS- or HA-repaired cartilage. The PACI + PRP treatment advanced the repair cartilage process in chondral defects with mature hyaline cartilage and enhanced the structural and mechanical qualities with better consistent cartilage, less susceptible to degradation and without hypertrophic formation over time.

## 1. Introduction

Articular cartilage is characterized by a hyaline structure composed of water, collagen, proteoglycan, and chondrocytes. Collagen is the main protein in the composition of the extracellular matrix (ECM), comprising approximately 60% of the dry weight of hyaline cartilage. Collagens provide tensile strength, regulate cell adhesion, support chemotaxis and migration, and direct tissue development [1,2].

Injuries in this tissue commonly cause knee pain and dysfunction. Additionally, these injuries may lead to early-onset osteoarthritis and have a huge negative impact on patients’ functions and quality of life [3]. Unfortunately, chondral lesions are difficult to treat because of their poor healing and regeneration potential [4,5]. Despite the improvement in technology and tissue engineering, it remains difficult to find an effective treatment for articular repair that is capable of restoring normal hyaline cartilage [4]. Minimal data are available regarding the intrinsic repair processes of damaged cartilage, and consequently, the search for a treatment that can restore normal hyaline cartilage is difficult. For this reason, it is critical to evaluate, in depth, the repair quality of repaired cartilage, including an exhaustive assessment of collagen types [6].

A possible treatment is the application of small, previously particulated or minced cartilage chips, described for the first time by Albrecht et al. [7]. The use of particulated autograft cartilage implantation (PACI) for the treatment of cartilage lesions relies on the migration of chondrocytes into a biomaterial and subsequent cartilaginous ECM deposition by these cells [8]. Platelet-rich plasma (PRP) has been proposed as a therapeutic option in musculoskeletal conditions given the important role of platelets in hemostasis, inflammation, and proliferation for tissue remodeling and healing [9]. Some studies have reported that PRP can stimulate chondrogenic regeneration, enhance the biosynthesis of cartilage matrix proteins, and enhance chondrocyte proliferation and metabolism [10]. In addition, PRP has been used in vivo in combination with other techniques to treat chondral defects, improving the quality of the repaired tissue [11].

In this study, a PACI + PRP therapeutic technique based on an autologous matrix composed of healthy hyaline cartilage chips included and mixed in a PRP clot, and an intraarticular infiltration of PRP was used to treat full-thickness chondral defects in the weight-bearing area of the medial femoral condyle of sheep. Two preliminary case reports provided excellent clinical, functional, and MRI-based outcomes in young active individuals with full-thickness cartilage or osteochondral defects treated with PACI + PRP [3,12]. Moreover, a preliminary study conducted on sheep treated with PACI + PRP after 1, 3, and 6 months of recovery revealed improvements in chondrogenesis and the regeneration of newly formed cartilage after 6 months of recovery [13]. However, these preliminary studies have certain limitations, such as a small sample size and a lack of a deep evaluation of the quality and organization of the ECM in the repaired cartilage tissue.

The appropriate zonal organization of the components of the ECM is a feature that distinguishes articular hyaline cartilage from other types of cartilage. Additionally, collagen and proteoglycan organization in adult hyaline articular cartilage is important for the maintenance of tissue load-bearing capacity [6,14,15]. Therefore, the goal of the present study is to perform an extensive and detailed immunohistochemical analysis of major and minor collagen types and aggrecan, allowing for the evaluation of the quality, durability, and structure of repaired cartilaginous ECM tissue using an autologous-based PACI + PRP treatment. We hypothesized that this treatment could restore durable and good-quality hyaline cartilage with an organized ECM.

## 2. Results

**Figure 1:**
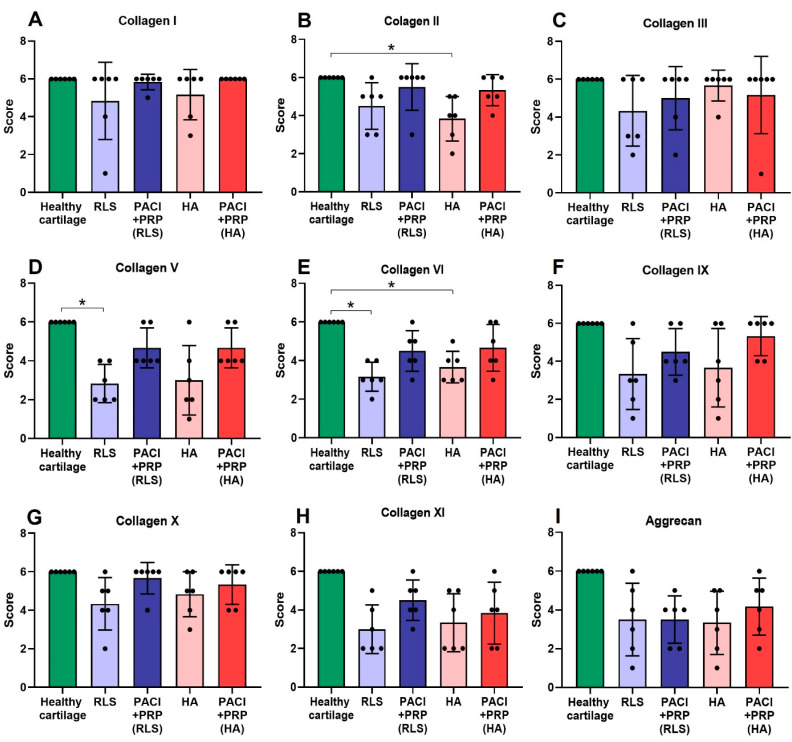
Immunostaining scores of collagen types and aggrecan studied for RLS, PACI + PRP(RLS), HA, and PACI + PRP(HA) at 9 months. (**A**) Type I collagen; (**B**) type II collagen; (**C**) type III collagen; (**D**) type V collagen; (**E**) type VI collagen; (**F**) type IX collagen; (**G**) type X collagen; (**H**) type XI collagen; (**I**) aggrecan. RLS: left knee treated with Ringer’s lactate solution; PACI + PRP(RLS): right knee treated with particulated autograft cartilage implantation and platelet-rich plasma from the group treated with RLS in the left knee; HA: left knee treated with hyaluronic acid; PACI + PRP(HA): right knee treated with particulated autograft cartilage implantation and platelet-rich plasma from the group treated with HA in the left knee. * Significant difference between groups, *p* < 0.05.

**Figure 2:**
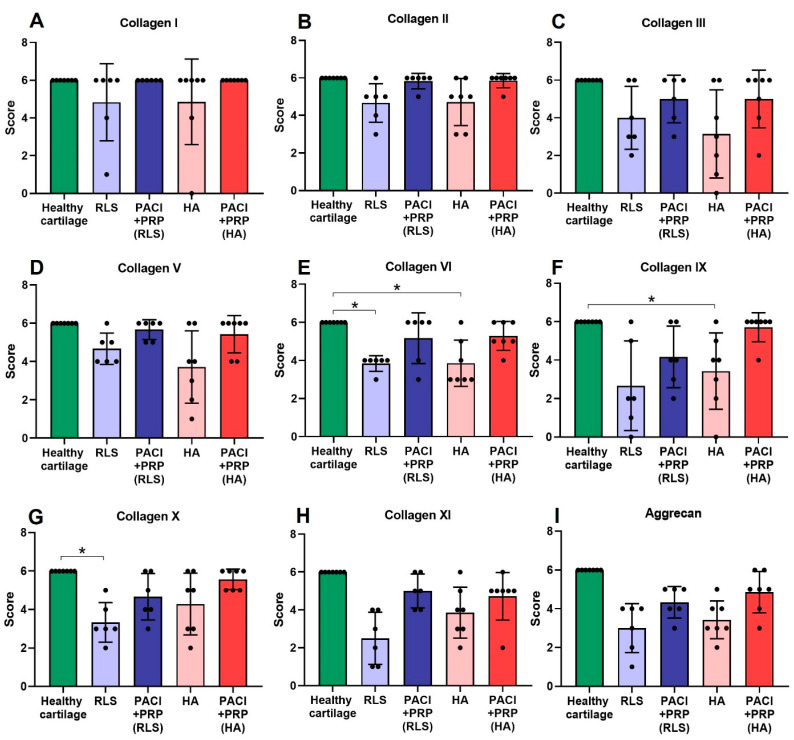
Immunostaining scores of collagen types and aggrecan studied for RLS, PACI + PRP(RLS), HA, and PACI + PRP(HA) at 18 months. (**A**) type I collagen; (**B**) type II collagen; (**C**) type III collagen; (**D**) type V collagen; (**E**) type VI collagen; (**F**) type IX collagen; (**G**) type X collagen; (**H**) type XI collagen; (**I**) aggrecan. RLS: left knee treated with Ringer’s lactate solution; PACI + PRP(RLS): right knee treated with particulated autograft cartilage implantation and platelet-rich plasma from the group treated with RLS in the left knee; HA: left knee treated with hyaluronic acid; PACI + PRP(HA): right knee treated with particulated autograft cartilage implantation and platelet-rich plasma from the group treated with HA in the left knee. * Significant difference between groups, *p* < 0.05.

As presented in Figure 1 and Figure 2, at 9 and 18 months, respectively, the score of the studied collagens and aggrecan obtained from the PACI + PRP groups (*n* = 25) were the closest to the surrounding healthy cartilage compared with the other study groups. In contrast, for some of the collagens (types II, V, VI, IX, and X) the RLS or HA groups scored significantly lower than the surrounding healthy tissue (Figure 1 and Figure 2) (Table 1).

The presence of type I collagen, both in normal hyaline cartilage and in the PACI + PRP groups, is confined to the bone and is completely absent in the cartilage. However, in the RLS and HA groups, some positivity for this collagen in the ECM can be observed at 9 and 18 months (Figure 1A and Figure 2A).

**Figure 3:**
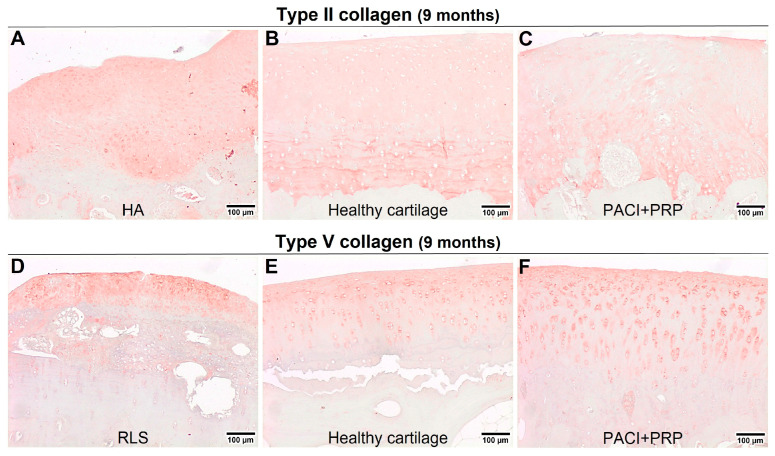
Immunohistochemical evaluation of healthy cartilage and repaired tissue at 9 months after the administered treatments. (**A**) Evaluation of type II collagen in HA group at 9 months; (**B**) example of healthy cartilage surrounding the chondral defect immunostained with type II collagen at 9 months; (**C**) evaluation of type II collagen in the PACI + PRP group at 9 months; (**D**) evaluation of type V collagen in the RLS group at 9 months; (**E**) example of healthy cartilage surrounding the chondral defect immunostained with type V collagen at 9 months; (**F**) evaluation of type V collagen in the PACI + PRP group at 9 months. RLS: left knee treated with Ringer’s lactate solution; HA: left knee treated with hyaluronic acid; PACI + PRP: right knee treated with particulated autograft cartilage implantation and platelet-rich plasma.

**Figure 4:**
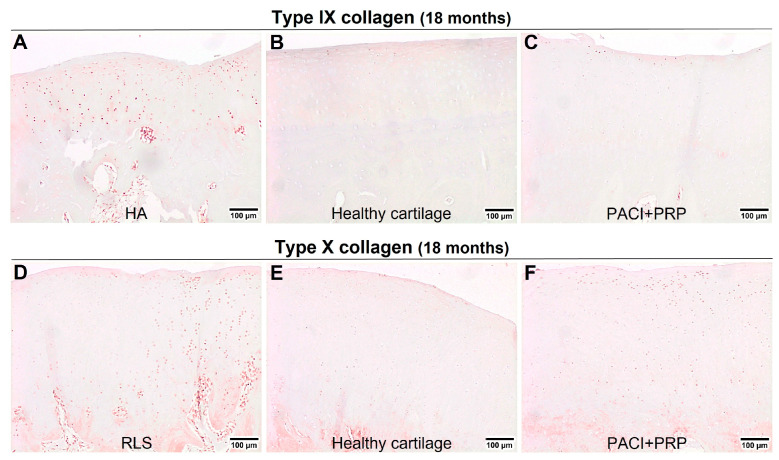
Immunohistochemical evaluation of healthy cartilage and repaired tissue at 18 months after the treatments administered. (**A**) Evaluation of type IX collagen in the HA group at 18 months; (**B**) example of healthy cartilage surrounding the chondral defect immunostained with type IX collagen at 18 months; (**C**) evaluation of type IX collagen in the PACI + PRP group at 18 months; (**D**) evaluation of type X collagen in the RLS group at 18 months; (**E**) example of healthy cartilage immunostained surrounding the chondral defect with type X collagen at 18 months; (**F**) evaluation of type X collagen in the PACI + PRP group at 18 months. RLS: left knee treated with Ringer’s lactate solution; HA: left knee treated with hyaluronic acid; PACI + PRP: right knee treated with particulated autograft cartilage implantation and platelet-rich plasma.

At 9 or 18 months, statistically significant differences were observed between healthy cartilage and the HA group for type II collagen (*p* = 0.03) (Figure 1B) or type IX collagen (*p* = 0.03) (Figure 2F), respectively. The HA group presented a higher percentage of type II (Figure 3A,B) and type IX (Figure 4A,B) collagens than the percentage found in healthy cartilage. In healthy cartilage and the PACI + PRP groups, type II collagen was found throughout the cartilage ECM (Figure 3B,C). In the HA group, type II collagen was observed throughout the ECM, although it was also observed with more intensity in the pericellular matrix of chondrocytes in the superficial, deep, and calcified zones (Figure 3A). Type IX collagen was observed in some chondrocytes of the superficial zone in healthy cartilage and the PACI + PRP groups (Figure 4B,C), while in the HA group, type IX collagen was identified in all chondrocytes (Figure 4A).

Both type V collagen at 9 months (*p* = 0.03) (Figure 1D) and type X collagen at 18 months (*p* = 0.03) (Figure 2G) showed statistically significant differences between healthy cartilage and the RLS group. The RLS group presented a higher percentage of type V (Figure 3D,E) and type X (Figure 4D,E) collagen than the healthy hyaline cartilage. Additionally, the pattern and intensity of immunostaining differed between the RLS group and healthy cartilage. In healthy cartilage and the PACI + PRP groups, type V collagen was found mainly in the pericellular matrix of chondrocytes and with decreasing intensity from the upper superficial to the bottom calcified zones (Figure 3E,F), while in the RLS group, type V collagen was found in the pericellular matrix of chondrocytes but also in the rest of the ECM, with much more immunostaining intensity (Figure 3D). Type X collagen was found in bone and in some superficial chondrocyte zones, as it was observed in healthy cartilage (Figure 4E). However, in the RLS group, the presence of type X collagen was observed in most of the chondrocytes in all the cartilage zones (Figure 4D).

**Figure 5:**
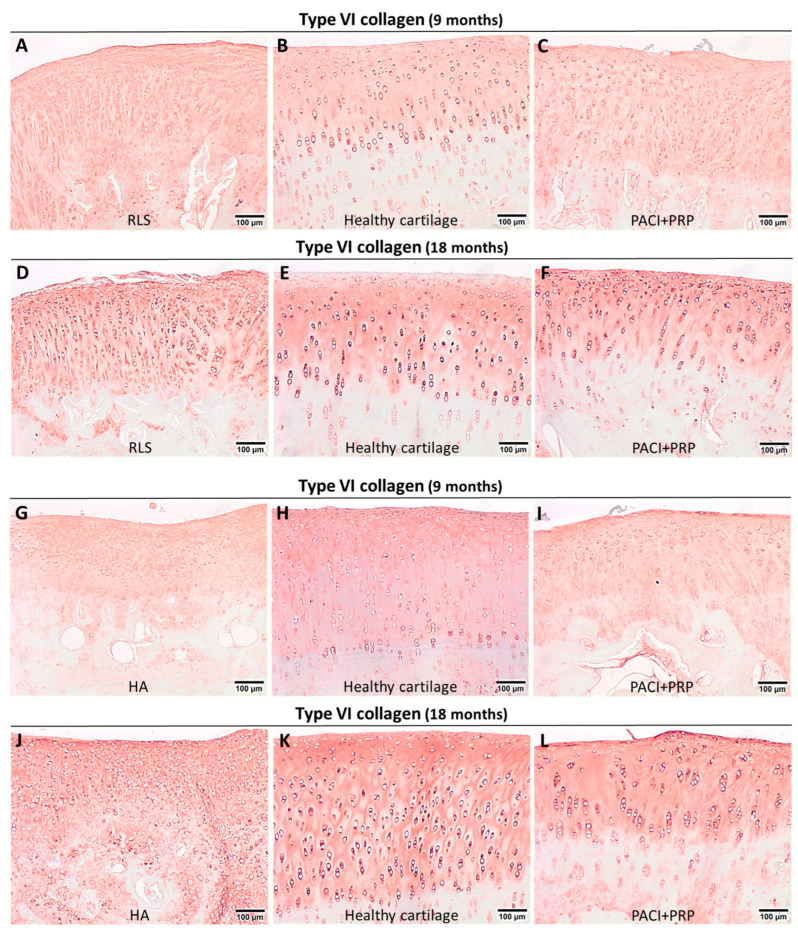
Immunohistochemical evaluation of healthy cartilage and the repaired tissue at 9 and 18 months after the treatments administered. (**A**) Evaluation of type VI collagen in the RLS group at 9 months; (**B**) example of healthy cartilage surrounding the chondral defect immunostained with type VI collagen at 9 months; (**C**) evaluation of type VI collagen in the PACI + PRP group at 9 months; (**D**) evaluation of type VI collagen in the RLS group at 18 months; (**E**) example of healthy cartilage surrounding the chondral defect immunostained with type VI collagen at 18 months; (**F**) evaluation of type VI collagen in the PACI + PRP group at 18 months; (**G**) evaluation of type VI collagen in the HA group at 9 months; (**H**) example of healthy cartilage surrounding the chondral defect immunostained with type VI collagen at 9 months; (**I**) evaluation of type VI collagen in the PACI + PRP group at 9 months; (**J**) evaluation of type VI collagen in the HA group at 18 months; (**K**) example of healthy cartilage surrounding the chondral defect immunostained with type VI collagen at 18 months; (**L**) evaluation of type VI collagen in the PACI + PRP group at 18 months. RLS: left knee treated with Ringer’s lactate solution; HA: left knee treated with hyaluronic acid; PACI + PRP: right knee treated with particulated autograft cartilage implantation and platelet-rich plasma.

Positivity for type VI collagen showed statistically significant differences between healthy cartilage and the RLS group (*p* = 0.03 and *p* = 0.03) and between healthy cartilage and HA-treated cartilage (*p* = 0.03 and *p* = 0.03), both at 9 and 18 months (Figure 1E and Figure 2E). Groups treated with RLS or HA presented a higher percentage of type VI collagen than the percentage found in healthy cartilage (Figure 5). In healthy cartilage, type VI collagen was found with great intensity in the pericellular matrix of all chondrocytes and in the rest of the ECM of the superficial and middle zones, and it was absent in the ECM of the deep and calcified zones (Figure 5B,E,H,K). At 9 months, a decrease in the intensity of immunostaining in the chondrocyte pericellular matrix was observed in the RLS- and HA-treated groups (Figure 5A,G). Additionally, at 9 months, type VI collagen was perceived in the ECM of the deep and calcified zones of the RLS group (Figure 5A). At 18 months, an improvement in the RLS group was detected related to the high intensity of immunostaining in the chondrocyte pericellular matrix, and type VI collagen was not observed in the ECM of the calcified zone. However, type VI collagen was already distinguished in the ECM of the deep zone (Figure 5D). In the HA group at 18 months, the intensity of the immunostaining in the pericellular matrix was stronger; however, type VI collagen was observed in the ECM of the deep and calcified zones (Figure 5J). The intensity of immunostaining in the pericellular matrix in the PACI + PRP groups was lower than in healthy cartilage at 9 months; however, type VI collagen distribution was the same as in healthy cartilage (Figure 5C,I), whereas, at 18 months, type VI collagen distribution and the intensity of immunostaining in the PACI + PRP groups was the same as healthy cartilage (Figure 5F,L).

## 3. Discussion

Hyaline articular cartilage is a highly specialized connective tissue, optimized to support and transmit loads with a low coefficient of friction. This, to a large extent, is attributed to the collagens present in the ECM [16]. However, given some of its distinctive features, such as the absence of blood vessels, lymphatic vessels, and nerves, as well as the fact that its cells have low mitotic potential, the repair capacity of cartilage is very limited [17,18]. Despite the large number of existing methods for the treatment of chondral lesions [4,17,19], it has not been possible to restore completely normal hyaline cartilage. An increasing number of studies support the potential of techniques based on particulated cartilage, such as PACI or minced cartilage implantation (MCI) [12,20,21]. The use of MCI or PACI for the treatment of chondral defects relies on the migration of chondrocytes into a biomaterial and subsequent cartilaginous ECM depositions by these cells [22]. Recent in vitro research that studied the repair process mechanisms behind PACI + PRP therapy showed that the cartilage fragments embedded in the three-dimensional PRGF scaffold contain viable chondrocytes that were able to migrate into the fibrin network, proliferate, and synthesize ECM [23]. The better quality of repaired tissue matrix has been shown in animal [13,22,24,25] and human [3,12,22] studies. Previously, a study was published where the chondrogenic regenerative properties of PACI + PRP were analyzed at a macroscopic and histological level. Improved macroscopic appearance, enhanced histological structure, and chondral repair were observed following the application of PACI + PRP as a treatment for chondral defects in sheep [25].

In this study, a therapy for chondral lesions based on a combination of treatment with PACI and PRP was investigated. This treatment was an entirely autologous therapy, and the surgery was performed in an on-step procedure, so the cost can be significantly reduced [20,21]. These cartilage chips and PRP were part of a bioactive matrix that conducted the repair process in the chondral defect. It is essential to take into account their degree of fragmentation since it correlates positively with the exposed surface area of the biomaterial in which the particles are embedded [26]. Bonasia et al. [27] recommended fragmenting the cartilage into cubes of about 2 mm^3^. In our study, the excised cartilage was fragmented into cubes of between 1 and 2 mm^3^. Another important parameter of PACI treatment is the scaffolds used to support the cartilage particles. Protein-based scaffolds such as collagen and fibrin provide binding sites for chondrocytes to adhere to, making them good candidates for scaffolding. Fibrin glue is one of the most commonly used; however, some of its components affect both mechanical strength and adhesive properties [28]. The use of alternative scaffolds has also been described. Marmotti et al. [24] carried out a study on rabbits in which they treated chondral defects using PACI on a scaffold composed of a derivate of HA, fibrin glue, and PRP. Furthermore, in vivo evaluation with PRP scaffolds promoted cell migration but also served as a bioactive scaffold in order to promote chondrocyte viability, proliferation, and differentiation, acting as a reservoir of growth factors and cytokines in [29]. The use of growth factors plays a key role in chondrogenesis during cartilage repair [30,31].

Minimal information is available regarding the repair processes of damaged cartilage, and consequently, there is increased difficulty in finding a treatment that can restore normal articular cartilage. For this reason, it is critical to evaluate, in depth, the repair quality of the repaired cartilage, including an exhaustive evaluation of collagen types. Numerous important collagen subtypes have been identified in healthy articular cartilage, which can be classified as main collagens (types II, IX, and XI) and minor collagens (types III, V, VI, and X) [6]. Additionally, it was determined that type I collagen can be identified in damaged or pathologic articular cartilage [32]. Studies that have evaluated the quality of chondral repair during their treatment have analyzed only some of these collagen types. For instance, Yvonne et al. [33] analyzed type I, II, and VI collagens; Dongquan et al. [34] analyzed type I and II collagens; and Wenqiang et al. [35] analyzed type I, II, and X collagens. However, in this study, we employed a scoring system that allowed us to analyze, in depth, these main and minor collagens, as well as type I and aggrecan, thus obtaining greater evidence on the quality and durability of the repaired tissue.

Type II and V collagens are fibril-forming collagens, characterized by periodic fibrils with an indeterminate length according to the stage of development [36]. In our results, at 18 months, we did not observe significant differences between healthy cartilage and the PACI + PRP, RLS, or HA groups for type II and V collagens. However, at 9 months, significant differences were detected between healthy cartilage and the HA group for type II collagen and between healthy cartilage and the RLS group for type V collagen, which indicated that, at this time, the RLS and HA groups did not reach a fully mature stage of development. These results showed that the PACI + PRP treatment generated mature hyaline cartilage in a shorter period of time than the RLS or HA groups.

Type IX collagen is a main collagen that stabilizes fibrillar networks by laterally associating with type II and type XI collagens. Type IX collagen reduction in articular cartilage produces a weaker ECM that is predisposed to degradation [37]. Type X collagen is specific to the calcified cartilage zone [38], is synthesized by hypertrophic chondrocytes, and is used as a marker of chondrocyte hypertrophy [39]. In our results, at 9 months, significant differences were not detected between healthy cartilage and the PACI + PRP, RLS, or HA groups for type IX and X collagens. However, at 18 months, significant differences were observed between healthy cartilage and the HA group for type IX collagen and the RLS group for type X collagen. These findings suggested that repaired cartilage tissue deteriorates over time in both the HA and RLS groups, being more susceptible to degradation and hypertrophic cartilage formation, respectively. Therefore, these results showed that the PACI + PRP treatment regenerated consistent cartilage without hypertrophic cartilage formation at the studied times.

Type VI collagen plays a key role in interactions between chondrocytes and ECM, contributing to the ECM’s structural integrity and mechanical properties [38]. In our research, significant differences were observed for type VI collagen between healthy cartilage and the RLS and HA groups at 9 and 18 months. In contrast, significant differences were not detected for this collagen in the PACI + PRP groups at either time point. Thus, the PACI + PRP treatment induced better structural and mechanical qualities in the repaired cartilage.

Sheep have been shown to be a viable option as an experimental model for translational investigation in new treatment options for knee joint injuries [40]. However, it is a very demanding animal model because rest is not possible. Postoperative rehabilitation is also impossible. Although in this study the results showed that the PACI + PRP treatment improved the quality of chondral repair, there was a large dispersion in the data obtained, without which, we suspect we could have obtained more significant results. This dispersion is probably due to the postoperative limitations encountered. Furthermore, these results could be even more promising when they are translated to humans, as it would be possible to carry out the necessary rest and subsequently carry out rehabilitation.

## 4. Materials and Methods

### 4.1. Ethical Statement

The present study was approved by the Bioethical Committee on Animal Research of the Regional Government Andalusia (Junta de Andalucía 12/06/2016/109—reference SSA/SIS/MD/jv) and conducted in accordance with guidelines for the protection of animals utilized for scientific purposes (Directive 2010/63/UE, Decision 2020/569/UE, and RD 1386/2018).

### 4.2. Study Design and Surgical Procedure

Twenty-eight skeletally mature and healthy Merino sheep (*n* = 28), weighing between 50 and 60 kg were used for this study.

A medial mini-arthrotomy of 4 cm was performed on both knees of the sheep. An 8 mm full-thickness cartilage defect was created in the weight-bearing area of the medial femoral condyle, and the cartilage was excised. The right hind limbs of the sheep (28 right knees) were treated with the PACI + PRP technique. The excised cartilage sample was sliced into small, 1–2 mm^3^ fragments and mixed with activated PRP to obtain a clot used as a scaffold for cartilage chips. The clot and cartilage chips were placed, filling the created cartilage defect. Then, it remained in a stationary position for 5 min to ensure the adherence of the clot to the defect. Following adherence, the surgical site was sutured, and 2 mL of activated PRP was intraarticularly injected. The left knees of the sheep were randomly divided to use two different control treatments. Subsequently, after performing the same surgical procedure of creating a chondral defect and closing the incision, half of the left knees received an intra-articular injection of 2 mL of Ringer’s lactate solution (RLS) (14 left knees), and the other half were treated with 2 mL of hyaluronic acid (HA) (Synvisc One, Hylan G-F 20) (14 left knees). Finally, antibiotic (amoxicillin–clavulanic acid, 10 mg/kg IM) and analgesic (buprenorphine 0.02 mg/kg/8 h IM) treatments were administered for three and five days after surgery, respectively. The animals were allowed to move freely without splints in an indoor stable.

Animals were randomly divided into two study groups: the RLS/PACI + PRP(RLS) group, which included fourteen sheep treated with RLS in the left knee and PACI + PRP in the right knee (*n* = 14), and the HA/PACI + PRP(HA) group, which included fourteen sheep treated using HA in the left knee and PACI + PRP in the right knee (*n* = 14). The time of sacrifice was initiated 9 or 18 months after surgery. Seven sheep from each study group were randomly sacrificed at the allotted time.

Throughout the study, three animals were lost: one sheep from the RLS/PACI + PRP(RLS) group at 9 months, one sheep from the HA/PACI + PRP(HA) group at 9 months, and one sheep from the RLS/PACI + PRP(RLS) group at 18 months.

### 4.3. Preparation and Use of Autologous PRP

The PRP treatment was prepared according to a previously reported method [16]. Blood was collected from the jugular vein of each animal in 5 mL collection tubes with 0.5 mL of sodium citrate solution (3.8%) as an anticoagulant and centrifuged over 8 min at 630× *g*. Blood was collected just prior to surgery and was processed intraoperatively. The plasma volume was divided into two (50%). The upper layer of centrifuged plasma was fraction 1, and the deeper layer of the centrifuged plasma just over of the buffy coat was fraction 2. Fraction 2, including a platelet concentration of 2- to 2.5-fold higher than peripheral blood, was obtained by pipetting with precision to avoid the aspiration of white and red blood cells. Platelets were activated by adding 50 μL of calcium chloride (10%) for 1 mL of plasma ratio just prior to use. To obtain the semisolid scaffold, including the cartilage chips, activated fraction 1 and fraction 2 were used in equal parts (50/50). For the intraarticular injection of 2 mL of PRP, only activated fraction 2 was used. The entire treatment included PACI + PRP application and an intraarticular PRP injection. The time delay between blood collection and both fractions’ use was less than 1.5 h. The PRP treatment was only applied intraoperatively with no postoperative doses administered.

### 4.4. Immunohistochemical Evaluation

An immunohistochemical study was used to assess collagen types (types I, II, III, V, VI, IX, X, XI) and proteoglycan (aggrecan) positivity in cartilage samples using the avidin–biotin–peroxidase method. After the sacrifice of each sheep, the medial femoral condyles were harvested from both knees and then immediately preserved in 10% buffered formaldehyde for 24 h, decalcified for 72 h (Thermo Scientific^TM^ Shandon TBD-1^TM^ Decalcifier, Cheshire, United Kingdom), and then processed and embedded in paraffin wax in the customary fashion. Sections of 4 μm were obtained for immunohistochemical staining. Each sample was coded to blind the analysis to the researchers. Samples were routinely processed and embedded in paraffin wax. Tissue sections were dewaxed and rehydrated, and endogenous peroxidase activity was exhausted via incubation with 0.3% hydrogen peroxidase (Panreac, Barcelona, Spain) in methanol (Panreac, Barcelona, Spain) at room temperature (RT). Two different antigen retrieval pre-treatments were used, heat-induced antigen retrieval with 0.01 M sodium citrate buffer pH6 (antibody to collagen type IX) and an enzymatic pre-treatment with hyaluronidase (antibodies to collagen types I, II, III, V, VI, X, XI and aggrecan), both using a laboratory oven at 50 °C for 45 min. Sections were washed in phosphate-buffered saline (PBS) at pH 7.2 for 10 min and incubated with 20% normal goat serum (MP Biomedicals, San Francisco, CA, United States) at RT for 30 min. A panel of primary antibodies was diluted in PBS containing 10% normal goat serum (Table 2) and incubated overnight at 4 °C. According to the manufacturer, all primary antibodies show negligible cross-reaction with non-specific collagen types, and all cross-react with several species of mammals, including sheep [16]. Following washing in PBS, the sections were incubated with biotinylated goat anti-rabbit or anti-mouse (Table 2) secondary antibodies (Dako, Agilent, Santa Clara, CA, United States; E0432 and E0433, respectively) diluted to 1:200 and 1:50, respectively, for 30 min at RT. After washing in PBS, the sections were incubated with ABC complex (Vector Laboratories, Burlingame, CA, United States) for 1 h at RT in darkness, washed in Tris-buffered saline pH 7.6, and then incubated in chromogen solution (Vector NovaRed Peroxidase Substrate Kit, Vector Laboratories, Burlingame, CA, United States). Finally, the sections were counterstained with Mayer’s hematoxylin and mounted with Eukitt (Freiburg, Germany). Tissue sections in which primary antibodies were substituted with non-immune serum were used as negative controls.

To determine the quality of the repaired tissue, chondral defects treated with RLS, HA, and PACI + PRP were immunohistochemically evaluated and compared with surrounding normal hyaline cartilage.

### 4.5. Development of a Semiquantitative Scoring System

The assessment system used in this study was based on previous publications [6,14,41,42,43]. In this scoring system, a semiquantitative assessment of immunostaining pattern and intensity was performed. Both assessed parameters were evaluated separately using a scale ranging from 0 to 3 (Table 3).

For this evaluation, the whole repaired tissue area was divided into four zones considering the physiological zones that comprised the healthy hyaline cartilage (superficial zone, middle or transition zone, deep zone, and calcified zone). Subsequently, to evaluate the structure of the repaired cartilage, the same zones were compared with repaired and normal cartilage tissue. First, when three of the zones compared yielded differences, the assigned score was 0 (severely abnormal). Second, when two zones differed, the score was 1 (abnormal). Third, when one zone differed, the score was 2 (nearly normal). Fourth, when no zones differed, the score was 3 (normal). Finally, a score from 0 to 6 points was obtained, where 6 points indicated that the repaired cartilage immunostaining was equal to that of the surrounding normal hyaline cartilage. This evaluation was graded blindly.

### 4.6. Statistical Analyses

Statistical analysis was performed using GraphPad Prism 7.0 (Inc., San Diego, CA, United States). First, a Kolmogorov–Smirnoff normality test was used to determine whether the studied variables were normally distributed. A Wilcoxon test was performed to compare treatments used on the same sheep at the same time of sacrifice, while a Mann–Whitney U test was carried out to compare treatments between different animals. In addition, a Mann–Whitney U test was performed to compare the two sacrifice periods (9 vs. 18 months after surgery) for the same treatment group. The variables in the tables were expressed as mean ± standard deviation. The variables were considered significant when *p* < 0.05 (* *p* < 0.05).

## 5. Conclusions

From an immunohistochemical point of view, the PACI + PRP treatment advanced the repair cartilage process in chondral defects with mature hyaline cartilage (related to type II and V collagens) and enhanced the structural and mechanical qualities (related to type VI collagen) with better consistent cartilage that was less susceptible to degradation (related to type IX collagen) and without hypertrophic formations over time (related to type X collagen).

## Figures and Tables

**Table 1:** Collagen types and aggrecan score results for all studied groups at 9 or 18 months.

Month	Variable	Group				
		HC	RLS	PACI + PRP (RLS)	HA	PACI + PRP (HA)
9	Col I	6 ± 0	6 ± 2.04	6 ± 0.41	6 ± 1.33	6 ± 0
	Col II	6 ± 0 ^b^	5 ± 1.22	6 ± 1.22	4 ± 1.17 ^b^	5.50 ± 0.82
	Col III	6 ± 0	4.50 ± 1.86	6 ± 1.67	6 ± 0.82	6 ± 2.04
	Col V	6 ± 0 ^a^	2.50 ± 0.98 ^a^	4 ± 1.03	2.50 ± 1.79	4 ± 1.03
	Col VI	6 ± 0 ^a,b^	3 ± 0.75 ^a^	4.50 ± 1.05	3.50 ± 0.82 ^b^	4.50 ± 1.21
	Col IX	6 ± 0	3 ± 1.86	4 ± 1.22	3.50 ± 2.06	6 ± 1.03
	Col X	6 ± 0	4.50 ± 1.37	6 ± 0.82	5 ± 1.17	6 ± 1.03
	Col XI	6 ± 0	4 ± 1.17	4.50 ± 1.05	4.50 ± 1.67	4.50 ± 1.83
	Aggrecan	6 ± 0	3.50 ± 1.87	3 ± 1.76	3.50 ± 1.26	4.50 ± 1.21
18	Col I	6 ± 0	6 ± 2.04	6 ± 0	6 ± 2.27	6 ± 0
	Col II	6 ± 0	5 ± 1.03	6 ± 0.41	6 ± 1.25	6 ± 0.38
	Col III	6 ± 0	3.50 ± 1.67	5.50 ± 1.27	3 ± 2.34	6 ± 1.53
	Col V	6 ± 0	4.50 ± 0.82	6 ± 0.52	4 ± 1.90	6 ± 0.97
	Col VI	6 ± 0 ^a,b^	4 ± 0.41 ^a^	6 ± 1.33	3 ± 1.21 ^b^	5 ± 0.75
	Col IX	6 ± 0 ^b^	2 ± 2.34	4 ± 1.60	4 ± 1.99 ^b^	6 ± 0.75
	Col X	6 ± 0 ^a^	3 ± 1.03 ^a^	4.5 ± 1.21	5 ± 1.60	6 ± 0.53
	Col XI	6 ± 0	4.5 ± 1.05	5± 0.89	5 ± 0.90	5 ± 0.69
	Aggrecan	6 ± 0	4.5 ± 1.47	5 ± 0.75	5 ± 1.38	5 ± 0.82

HC: healthy cartilage; RLS: left knee treated with Ringer’s lactate solution; PACI + PRP(RLS): right knee treated with particulated autograft cartilage implantation and platelet-rich plasma from the group treated with RLS in the left knee; HA: left knee treated with hyaluronic acid; PACI + PRP(HA): right knee treated with particulated autograft cartilage implantation and platelet-rich plasma from the group treated with HA in the left knee. ^a^ Significant difference (*p* < 0.05) between healthy cartilage and RLS; ^b^ significant difference (*p* < 0.05) between healthy cartilage and HA.

**Table 2:** Details of primary-antibody anti-collagen types and anti-aggrecan.

Target	Source	Reference	Host/Clonality	Dilution	Host Species
Collagen I	Abcam φ	ab34710	Rabbit polyclonal	1/200	Rabbit
Collagen II	Abcam φ	ab34712	Rabbit polyclonal	1/100	Rabbit
Collagen III	Abcam φ	ab7778	Rabbit polyclonal	1/200	Rabbit
Collagen V	Abcam φ	ab7046	Rabbit polyclonal	1/200	Rabbit
Collagen VI	Abcam φ	ab6588	Rabbit polyclonal	1/1000	Rabbit
Collagen IX	Abcam φ	ab134568	Rabbit polyclonal	1/200	Rabbit
Collagen X	Abcam φ	ab49945	Mouse monoclonal	1/500	Mouse
Collagen XI alpha 2	Abcam φ	ab196613	Rabbit polyclonal	1/50	Rabbit
Aggrecan	Abcam φ	ab3778	Mouse monoclonal	1/100	Mouse

φ Abcam, Cambridge, United Kingdom.

**Table 3:** Immunohistochemical scoring system.

Parameters		Points	Final Score
Immunostaining Pattern	Severely abnormal	0	6
	Abnormal	1	
	Nearly normal	2	
	Normal	3	
Immunostaining Intensity	Severely abnormal	0	
	Abnormal	1	
	Nearly normal	2	
	Normal	3	

## Data Availability

The data presented in this study are available on request from the corresponding author.

## Abbreviations

ECM	Extracellular matrix
HA	Hyaluronic acid
MCI	Minced cartilage implantation
PACI	Particulated autograft cartilage implantation
PACI + PRP	Therapeutic technique based on an autologous matrix composed of healthy hyaline cartilage chips included and mixed in a PRP clot and intraarticular infiltration of PRP
PBS	Phosphate-buffered saline
PRP	Platelet-rich plasma
RLS	Ringer’s lactate solution
RT	Room temperature
